# *MTHFR* C677T is not associated with autism spectrum disorder in a Mexican cohort

**DOI:** 10.3389/fpsyt.2025.1452940

**Published:** 2025-11-17

**Authors:** Mirna Edith Morales-Marín, Omar Náfate-López, Amalia Guadalupe Gómez-Cotero, Miguel Angel Cid-Soto, Humberto Nicolini, Xochitl Helga Castro-Martínez

**Affiliations:** 1 Laboratorio de Genómica de las Enfermedades Psiquiátricas y Neurodegenerativas, Instituto Nacional de Medicina Genómica, Mexico City, Mexico; 2 Unidad de Neuropsiquiatria Infantil, "Dr Manuel Velasco Suárez”, Hospital de Especialidades Pediátricas del Centro Regional de Alta Especialidad, Tuxtla Gutierrez, Chiapas, Mexico; 3 Centro de Investigación en Ciencias de la Salud, Unidad Santo Tomás, Instituto Politécnico Nacional, Mexico City, Mexico; 4 Consorcio de Oncogenómica, Instituto Nacional de Medicina Genómica, Mexico City, Mexico

**Keywords:** *MTHFR*, C677T, genetic variant, ASD, Mexican population

## Abstract

Using genetic approaches to study autism spectrum disorder (ASD) is essential to understanding the etiology of the condition. The C677T variant has emerged as a risk factor, and here we present the first association study of this variant in a Mexican population with ASD. Our objective was to assess the variant *MTHFR* C677T (rs1801133) in a group of Mexican patients with ASD through a case-control association analysis. We found no significant association of *MTHFR* C677T and ASD, with no rate differences between cases and controls (C vs T: odds ratio = 0.9698, 95% confidence interval = 0.7773–1.21, *P* = 0.7858). Results of this and other studies evaluating the link between ASD and this variant have been controversial. Our findings suggest that other ancestry-related factors may play a role.

## Introduction

Autism spectrum disorder (ASD) is described by the *Diagnostic and Statistical Manual of Mental Disorders*, 5th ed., as a group of neurodevelopmental disorders characterized by repetitive patterns of behavior and difficulties in establishing communication and social interaction. The global prevalence is 1:160 live births, and ASD can be classified into different levels of severity based on previously described activities or interests (1, 2). ASD is a group of entities that is increasing, the CDC has reported a prevalence of 1:36 children have ASD at the age of 8 years (3). A single study has been reported in Mexico with epidemiological data on this condition, with a prevalence of 1:116 individuals in the City of León, Guanajuato (4).

This group of disorders is called a spectrum because of the range of symptoms and their intensity and co-occurrence or not with intellectual disability. Symptoms begin to manifest at very early ages. The exact etiology remains poorly understood but involves both genetic and environmental factors (5). Several risk factors have been described, including paternal age, intrauterine exposure to maternal infection or some medications, intestinal microbiota profile, cesarean section, environmental contaminants, among others (6). As well as certain protective factors such as the intake of vitamin D, folic acid, breastfeeding (6–8). Early detection of ASD and timely intervention are important for supporting personal development, facilitating independence, and reducing family and socioeconomic impact. Currently, no molecular diagnostic testing is available for ASD, and diagnosis is made using psychiatric scales.

The human Methylenetetrahydrofolatereductase gene (*MTHFR*), located in chromosome region 1p36.3, encodes an enzyme involved in folate metabolism. Several single nucleotide variants (SNVs) on *MTHFR* have been linked to enzyme defects and different human diseases, including occlusive vascular disease, neural tube defects, colon cancer, and acute leukemia (9–12). Decreased MTHFR protein activity may affect the one-carbon metabolism cycle, which in turn might impair cortical and hippocampal neurogenesis during development and affect brain maturation and function (13). It has been reported that patients with ASD show a deficiency of the enzyme methylenetetrahydrofolate reductase and that after supplementation with traditional psychopharmacological treatment they had improvement in symptoms (14). The C677T SNV, among the most studied and clinically significant *MTHFR* variants, is located in exon 4 and involves the replacement of a cytosine by a thymine, leading to the substitution of valine for alanine at codon 222 (Zhang YX et al., 2022). Although previous reports have found an association of C677T SNVs with ASD (15–19) (Pu D et al., 2013; Rai V 2016; Wei H et al., 2021; Li Y et al., 2020; Sadeghiyeh T et al., 2019), most of these studies have been performed in populations with European or Asian ancestry, and admixed Latino American populations are strongly underrepresented. To bridge this gap, here we evaluated the association of the C677T SNV with ASD in a Mexican population.

## Material and methods

### Sample population

The study included 179 patients with ASD selected through the CMIGA consortium (Consorcio Mexicano de Investigación en Genética del Autismo) (20) and 1483 unaffected individuals. ASD participants were ages 2–18 years, and their parents agreed to their participation in the study. All ASD participants and their parents underwent an Autism Diagnostic Interview (21) and signed an informed consent and assent for minors. The control group was composed of 1483 individuals, which belong to the MxGDAR (Mexican Genomic Database for Addiction Research) cohort. This cohort is an epidemiological sample composed of 3393 healthy individuals recruited in Mexico from the 2016 National Survey of Drug, Alcohol, and Tobacco Use; where the 1483 individuals used as control subjects showed nor psychiatric symptoms neither consumption of risky substances (22). Thus, the use of this 1483 samples as control group discard any bias introduce if apparently healthy subjects were used as control group. Although the MxGDAR cohort overrepresented female individuals, our analysis showed no significant differences in the genotype and allele frequencies of rs1801133 variant. This study was performed in accordance with the Declaration of Helsinki and approved by the Ethics and Research Committee at the INMEGEN. The distribution of cases and controls is shown in **Table 1**.

**Table 1:** Distribution of cases and controls.

Samples	Gender	Mean age (years)	Diagnosis
Cases (179)	M (85.80%)	6.7	ASD
	F (14.20%)	5.8	ASD
Controls (1483)	M (27.38%)	30.20	None
	F (72.62%)	37.96	None

### DNA extraction and genotyping

Genomic DNA was extracted from blood lymphocytes or mouth swab using a commercial kit following the manufacturer’s protocol (Qiagen, Puregene). The genotype of the *MTHFR* C677T polymorphism in the studied sample was obtained from genotyping data generated with the Illumina Infinium^®^ PsychArray-24 v1.0 microarrays (Illumina, San Diego, California, USA) in the cohorts MxGDAR and CMIGA. This array contains 593,260 gene variants associated with predisposition and risk for developing psychiatric or neurodevelopmental conditions such as autism.

### Statistical analysis

Statistical analysis to assess the relationship between *MTHFR* C677T and ASD was performed using Plink v1.07 software (23). The Chi square test was used for group comparison between ASD participants and unaffected controls. Odds ratios and 95% confidence intervals were calculated, and *p* < 0.05 was set as indicating significance.

## Results

The most frequent genotype for both groups was CT, at 49% for ASD participants and 46% for controls (**Table 2**). No significant differences were observed between the groups for the *MTHFR* C677T variant (C vs T: OR = 0.9698, 95% CI = 0.7773–1.21, *p* = 0.7858) (**Table 3**). The control group results were consistent with Hardy–Weinberg equilibrium for the examined gene variant (*p* = 0.01219).

**Table 2:** *MTHFR* C677T genotype and allelic distribution in patients with ASD and control groups.

*MTHFR* C677T	Control N=1483 *(freq)*	Female n=1077 *(freq)*	Male n=406 *(freq)*	*P value*	Cases N=177 *(freq)*	Female n=22 *(freq)*	Male n=155 *(freq)*	*P value*
CC CT TT	345 *(0.23)* 690 *(0.47)* 448 *(0.30)*	249 *(0.23)* 488 *(0.45)* 340 *(0.32)*	97 *(0.24)* 201*(0.49)* 108 *(0.27)*	0.17234	37 *(0.21)* 88 *(0.50)* 52 *(0.29)*	3 *(0.14)* 14 *(0.64)* 5 *(0.22)*	33 *(0.21)* 75 *(0.49)* 47 *(0.30)*	1.00000
C T	1380 *(0.47)* 1586 *(0.53)*	986 *(0.46)* 1168 *(0.54)*	395 *(0.49)* 417 *(0.51)*	0.17252	162 *(0.46)* 192 *(0.54)*	20 *(0.45)* 24 *(0.55)*	141 *(0.45)* 169 *(0.55)*	1.00000

*P value* between gender for each group.

**Table 3:** Comparation of C677T allele frequency between ASD individuals and healthy controls.

Alleles	Control N=1483 *(freq)*	Cases N=177 *(freq)*	*P value*	OR	95% CI
C T	1380 *(0.47)* 1586 *(0.53)*	162 *(0.46)* 192 *(0.54)*	– 0.79	– 0.97	– 0.7773 - 1.21

OR, odds ratio; CI, confidence interval.

## Discussion

A recent meta-analysis comprising 2609 individuals with ASD and 7496 unaffected participants from 15 previously published studies found a significant association between the *MTHFR* C677T variant and ASD in all genetic models evaluated (*i.e.*, allelic, heterozygote, homozygote, dominant, and recessive), highlighting this variant as an important genetic risk factor for ASD (18). That meta-analysis included populations of European, East Asian, South Asian, Latin American, and African ancestry. An independent meta-analysis found a significant association of the *MTHFR* C677T variant with ASD in populations of European ancestry and in a population with Asian ancestry (16). Another meta-analysis evaluating the association of six candidate genes with ASD (*MTHFR, SLC25A12, OXTR, RELN, 5-HTTLPR*, and *SHANK*) identified the C677T variant as a consistent risk factor for ASD (17).

Similar to our findings, however, a previous study in a Han Chinese population of 201 children with ASD and 200 unaffected controls showed no significant association between *MTHFR* C677T and ASD or ASD severity (24). Likewise, this variant was not significantly associated with ASD in two independent studies in children, one with 151 ASD participants and 100 unaffected individuals in Brazil, and the other with 98 ASD participants and 70 unaffected individuals in Turkey (25, 26).

The frequency of the C677T variant in public genomic databases such as dbSNP changes depending on ethnic group. Its frequency in admixed Latino American groups is 0.45, whereas in the European population it is 0.34, and in South Asian individuals, it is 0.16 (27). Allele frequency can be modified by environmental and geographical factors, and ASD has a multifactorial etiology, so that the association of the C677T variant with ASD could be strongly dependent on other factors related to the ancestry of the population. Likewise, Kang J. et al., 2024 observed a genetic correlation between inflammatory bowel disease and osteoporosis in European populations but not in East Asian populations (28). Taking together, these findings indicate that the presence of other genetic factors, including genetic dominance, pleiotropy, fitness trade-offs, sign epistasis, and genetic linkage between loci interfere in the association of genetic variants with pathological conditions. Supporting this hypothesis, a study carried out on Mexican Mestizos and a diverse group of Native Americans from Mexico reported a frequency similar to that observed in our analysis, as well as the highest frequency worldwide for the variant allele in Mexican Amerindians (29). An important limitation of our study is the small sample size, and our findings must be validated using a larger population.

## Data Availability

The data presented in the study are deposited in the EVA repository, accession number PRJEB101865.
